# Appearances of screen-detected versus symptomatic colorectal cancers at CT colonography

**DOI:** 10.1007/s00330-016-4293-7

**Published:** 2016-04-05

**Authors:** Andrew A. Plumb, Fiona Pathiraja, Claire Nickerson, Katherine Wooldrage, David Burling, Stuart A. Taylor, Wendy S. Atkin, Steve Halligan

**Affiliations:** 1 Centre for Medical Imaging, University College London, London, UK; 2 Public Health England, Fulwood House, Sheffield, UK; 3 Department of Surgery and Cancer, Imperial College London, London, UK; 4 Intestinal Imaging Centre, St Mark’s Hospital, Harrow, UK

**Keywords:** Colorectal neoplasms, CT colonography, Mass screening, Occult blood, Computer-assisted diagnosis

## Abstract

**Objectives:**

The aim of this study was to compare the morphology, radiological stage, conspicuity, and computer-assisted detection (CAD) characteristics of colorectal cancers (CRC) detected by computed tomographic colonography (CTC) in screening and symptomatic populations.

**Methods:**

Two radiologists independently analyzed CTC images from 133 patients diagnosed with CRC in (a) two randomized trials of symptomatic patients (35 patients with 36 tumours) and (b) a screening program using fecal occult blood testing (FOBt; 98 patients with 100 tumours), measuring tumour length, volume, morphology, radiological stage, and subjective conspicuity. A commercial CAD package was applied to both datasets. We compared CTC characteristics between screening and symptomatic populations with multivariable regression.

**Results:**

Screen-detected CRC were significantly smaller (mean 3.0 vs 4.3 cm, p < 0.001), of lower volume (median 9.1 vs 23.2 cm^3^, p < 0.001) and more frequently polypoid (34/100, 34 % vs. 5/36, 13.9 %, p = 0.02) than symptomatic CRC. They were of earlier stage than symptomatic tumours (OR = 0.17, 95 %CI 0.07-0.41, p < 0.001), and were judged as significantly less conspicuous (mean conspicuity 54.1/100 vs. 72.8/100, p < 0.001). CAD detection was significantly lower for screen-detected (77.4 %; 95 %CI 67.9-84.7 %) than symptomatic CRC (96.9 %; 95 %CI 83.8-99.4 %, p = 0.02).

**Conclusions:**

Screen-detected CRC are significantly smaller, more frequently polypoid, subjectively less conspicuous, and less likely to be identified by CAD than those in symptomatic patients.

***Key Points*:**

• *Screen-detected colorectal cancers (CRC) are significantly smaller than symptomatic CRC.*

• *Screening cases are significantly less conspicuous to radiologists than symptomatic tumours.*

• *Screen-detected CRC have different morphology compared to symptomatic tumours (more polypoid, fewer annular).*

• *A commercial computer-aided detection (CAD) system was significantly less likely to note screen-detected CRC.*

**Electronic supplementary material:**

The online version of this article (doi:10.1007/s00330-016-4293-7) contains supplementary material, which is available to authorized users.

## Introduction

Computed tomographic colonography (CTC) is the radiological investigation of choice for suspected colorectal neoplasia because it is highly sensitive for colorectal cancer (CRC) [1] and adenomas ≥6 mm [2–6]. CTC is commonly applied in two distinct patient groups–firstly, those with symptoms suggesting CRC; and secondly, asymptomatic subjects undergoing screening. International consensus recommends CTC as suitable for investigation of symptomatic patients [7, 8]; a recent multicenter randomized trial showed no significant difference in detection rates of CRC and polyps ≥10 mm between CTC and colonoscopy [9]. Regarding screening, diagnostic sensitivity of 90 % for CRC and polyps ≥10 mm was achieved in one large, prospective, multicenter study [10], and a randomized trial found higher participation rates at screening CTC (vs. colonoscopy) [11] that translated to higher per-invitee advanced adenoma detection rates once surveillance procedures were included [12]. CTC can also be employed in screening programs using fecal occult blood testing (FOBt) because it is highly sensitive in this specific scenario [13]. In such cases, colonic imaging (usually colonoscopy) is reserved for those testing positive to FOBt—CTC is recommended by international consensus when colonoscopy is incomplete or not feasible [7, 8].

Although CTC is now disseminated widely, the comparative morphological features of screen-detected and symptomatic cancers have received little attention. Epidemiological series show that screen-detected cancers are typically of earlier stage than non-screen detected tumours [14] and have superior survival [15]. Similarly, cancers detected by screening CTC are generally of earlier histological stage than the population average [16]. Intuitively, symptomatic cancers and those detected by screening are likely to have different morphology at CTC—for example, screening cases being smaller and/or more subtle, although this is unproven. Additionally, although computer-assisted detection (CAD) is designed to detect polyps rather than cancer, many cancers have polypoid features and are therefore marked by CAD systems [17]. The relative performance of standalone CAD in screen-detected versus symptomatic tumours is also unknown.

In this study we aimed to document and compare the morphology, radiological stage, subjective conspicuity, and CAD characteristics of cancers detected by CTC in both FOBt screening and symptomatic populations.

## Materials and methods

### Permissions

CTC images depicting CRC derived from two sources: prospective collection via two paired, randomized trials (the Special Interest Group in Gastrointestinal and Abdominal Radiology [SIGGAR] trials); and retrospective collection from a national CRC screening program (the English Bowel Cancer Screening Programme, EBCSP). Ethical permission was granted for use of CTC data for future research in the randomized trials and waived by the Joint Research Office of the chief investigator for the screening datasets. Patients in the randomized studies gave written informed consent.

### Patient selection

#### Symptomatic cases

The SIGGAR trials were parallel, multicenter randomized trials of CTC versus barium enema and CTC versus colonoscopy in symptomatic patients. Patients aged ≥55 years were recruited at 21 hospitals following referral for the investigation of symptoms suggestive of CRC. The primary outcomes have been published elsewhere [9, 18], and focused on detection rates of CRC and polyps ≥10 mm [18]; and referral rates for further testing [9]. No data relating to CTC morphology of CRC diagnosed in either trial have been reported previously. For the current study, a trial statistician (KW) identified all patients who had (a) undergone CTC and (b) were diagnosed with CRC within the trial. CRC was defined as invasion of tumour cells into the submucosa or beyond. Anonymized CTC images were available for 35 patients (derived from 10 different centers), depicting 36 cancers. The images of 62 further patients (with 63 tumours) had not been returned to the trial office for review.

#### Screening cases

The EBCSP is a national FOBt-based screening program of adults aged 60–74 years. CTC is used when colonoscopy is incomplete or judged unsuitable. We used the program database to identify all individuals screened from April 2006 to March 2014 who (a) underwent CTC as their first colonic investigation after a positive FOBt result and (b) were ultimately diagnosed with CRC. We excluded individuals undergoing colonoscopy prior to CTC, since many are requested for colonic imaging upstream of an obstructing tumour, introducing spectrum bias toward larger/stenotic tumours. The images of 98 patients depicting 100 cancers (from 25 different centers), were transferred to the study office; images of a further 132 patients were requested but not received.

#### Sample size considerations

The sample size and power calculations for the primary endpoints of the randomized trials have been published previously [19]. The number of CRC in the symptomatic arm was dependent on this and therefore fixed. For screening cases, we assumed a mean tumour length and standard deviation of 3.0 and 1.6 cm, respectively [16], and aimed to estimate tumour length with a confidence interval of 1.5 cm. Using the approximation N = 4*σ^2^*(Z_crit_)^2^/D^2^, where N = sample size, σ = standard deviation, Z_crit_ = desired significance criterion, in this case 1.96 for 5 % significance, and D = desired confidence interval width [20], we required 70 screening cases. We allowed a 20 % increase for non-normality (i.e., 84 cases). We ceased attempting to retrieve more screening cases after this number had been reached (ultimately, more discs were received than anticipated).

### CTC imaging procedures

The RCTs required full bowel purgation for CTC, multidetector acquisition at ≥2.5 mm collimation and dual patient positioning. Oral fecal tagging agents were discretionary, as was the use of intravenous contrast. The EBCSP requires multidetector-row CTC at slice thickness of 1–3 mm and dual positioning. Fecal tagging, antispasmodics, and the use of carbon dioxide were recommended at program inception and mandated since 2012. Intravenous contrast is generally discouraged unless there is a specific requirement for detailed extracolonic evaluation. Detailed acquisition parameters are provided in Supplementary Table 1.

### Readers and viewing conditions

CTC dataset order was randomized using the *sample* command in R version 3.0.1 (R Foundation for Statistical Computing, Vienna, Austria). Subsequently, they were uploaded to both a commercial CTC workstation (Vitrea, Vital Images, Zoetermeer, The Netherlands) and an open-source DICOM viewer (Osirix, Pixmeo SARL, Bernex, Switzerland). Both two-dimensional and three-dimensional (i.e., endoluminal) displays were available on both platforms and used in the majority of cases.

Two radiologists (AAP, seven years experience of CTC) and (FP, two years experience) reviewed the CTC images independently. Each radiologist used the prone and supine images (with multiplanar reformatting and endoluminal views when needed) to record: (a) tumour morphology; either non-polypoid or polypoid; (b) for polypoid lesions, their sub-type using the Paris classification [21]: sessile, pedunculated, semi-pedunculated or flat (<2.5 mm height); (c) for non-polypoid lesions, whether they were annular (≥90 % of the colonic circumference) or non-annular; (d) presence/absence of luminal stenosis (≥50 % diameter reduction versus the immediately distal colonic segment); (e) tumour dimensions (maximum multiplanar long axis and orthogonal short axis for polypoid lesions; long axis and tumour thickness for non-polypoid tumours); (f) radiological T stage, using the TNM 7th edition [22]; (g) for T3 and T4 lesions, extramural depth of spread (EMD) beyond the muscularis propria; (h) presence/absence of radiologically-involved lymph nodes; (i) presence/absence of vascular invasion; (j) subjective image quality using a combined assessment of bowel cleansing and distension (1 = good, 2 = moderate or 3 = poor); (k) use of fecal tagging; and (l) subjective conspicuity of the tumour on a 100-point scale (1 = “barely visible”; 100 = “immediately obvious”). We followed the criteria of Burton et al. when determining lymph node involvement and vascular invasion [23] (nodal involvement=at least one node ≥1 cm in short axis, or a cluster of ≥3 nodes within the tumour local vascular pedicle; vascular invasion=nodular enlargement of colic veins).

Tumour segmental location was extracted from either SIGGAR trial or EBCSP records and confirmed from the images by both radiologists. Differences between radiologists regarding tumour morphology, T stage, EMD, nodal status, and vascular invasion were resolved by face-to-face discussion with images available, although we also recorded each radiologists’ original opinion for assessment of interobserver agreement. Since TNM stage does not always influence pre-operative treatment decisions, we also coded all tumours as either good-prognosis or poor-prognosis tumours, which is both reproducible and important when considering the administration of neoadjuvant therapy [24–26]. Specifically, tumours of either T1, T2, or T3 stage with <5 mm of EMD are regarded as having a good prognosis, whereas T4 and T3 tumours with either (a) radiologically-involved lymph nodes or (b) ≥5 mm of EMD are viewed as having a poor prognosis [25, 26].

Since radiologist opinions are inherently subjective, we generated objective measures of tumour location, ease of detection, and volume. To achieve this, AAP recorded (a) workstation-derived distance along the colonic centerline from the anorectal junction to the distal edge of the tumour; (b) presence/absence of at least one CAD mark within 5 mm of the tumour in any direction; (c) the total number of CAD marks for each patient; and (d) tumour volume, calculated by manually outlining the tumour on each slice and using the workstation’s volume calculation function. The CAD package used was iCAD (Nashua, New Hampshire) version 1.4.1.

### Statistical analysis

Data were collated using Microsoft Excel 2011 (Microsoft, Redmond, WA) and analyzed with R version 3.0.1. Since three patients had more than one tumour, analysis was on a per-patient basis for patient-level variables (e.g., scan quality, demographics) and on a per-lesion basis for tumour-level variables (e.g., dimensions, conspicuity).

Patient age and subjective image quality (averaged between readers) were compared between symptomatic and screening groups with the Mann-Whitney-Wilcoxon test. Patient sex and use of fecal tagging were compared using chi-squared and Fisher exact tests, respectively. To determine imaging features that differed significantly between symptomatic and screen-detected tumours after adjustment for age and sex, we applied multivariable regressions using linear, binary logistic, multinominal, or ordered logistic regression as appropriate. Tumour dimensions, volumes, and conspicuity scores were log-transformed to approximate normality prior to modeling. We used case origin (i.e., symptomatic vs screening) as the explanatory variable and patient age and sex as covariates. Image quality was added as a covariate for analysis of subjective conspicuity.

Agreement between radiologists’ initial independent reads for tumour stage, nodal stage, and presence of vascular invasion was calculated using quadratic weighted kappa for tumour stage and unweighted kappa for nodal and vascular stage. Probability values of <0.05 were taken as statistically significant.

## Results

### Patient characteristics and CTC image quality

Overall, 133 patients were included; 98 screening (34 female) with 100 tumours and 35 symptomatic (19 female) with 36 tumours. Patients in the EBCSP were younger than those in the RCTs (mean age: 68.2 years versus 71.9, p = 0.02). Subjective image quality was not significantly different between the two groups (screening patients, mean score: 1.5 out of 3; symptomatic patients: 1.4, p = 0.35). Fecal tagging was used for 72/98 patients in the screening program (73.5 %) but not for any patients in the randomized trials (0/35 = 0.0 %,p < 0.001).

### Imaging features of tumours

#### Segmental location and distance from the rectum

**Fig. 1:**
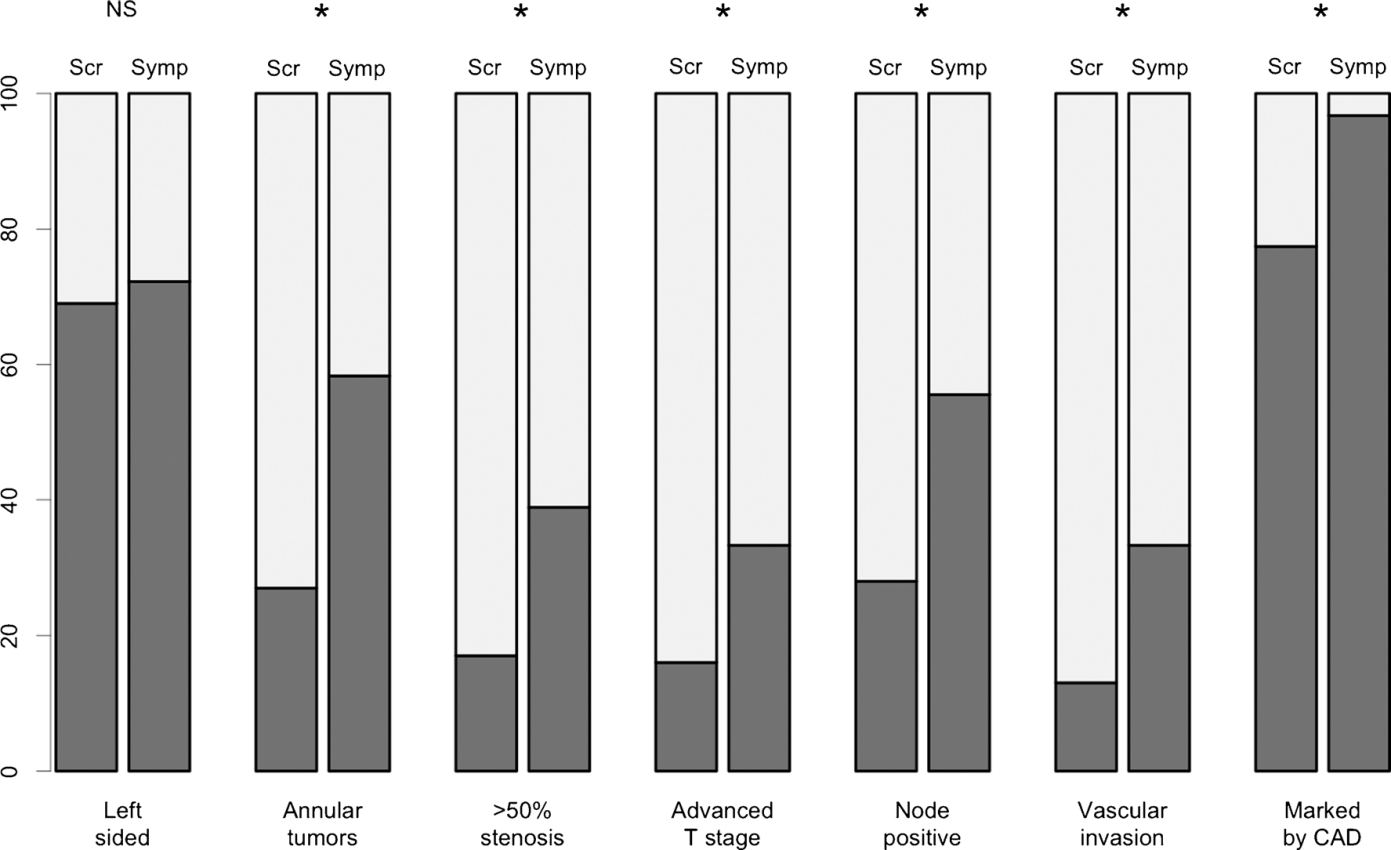
Bar charts showing tumour features that were recorded as binary variables; all charts show the percentage of tumours with (dark grey) or without (light grey) a given imaging feature. Asterisks indicate statistical significance at the 5 % level. “Advanced T stage” refers to either a T4 or T3 tumour with ≥5 mm of extramural spread. Scr = screen-detected tumours, Symp = symptomatic tumours

Most tumours were left-sided in both groups (screening: 69/100 tumours, 69.0 %; symptomatic: 26/36 tumours, 72.2 %,OR = 0.78, 95 %CI 0.36-1.69, p = 0.52, Table 1, Fig. 1). There was no significant difference in mean distance along the colonic centerline to the tumour (screening patients: mean = 68.5 cm; symptomatic patients: mean = 66.3 cm, p = 0.45).

**Table 1:** Morphology, dimensions, and subjective conspicuity of symptomatic and screen-detected cancers. Percentages use the number of tumours of that category (i.e., symptomatic or screening) as the denominator

	Screen-detected tumours (n = 100)	Symptomatic tumours (n = 36)	p value
**Location**			**0.52**
Left-sided (%)	69 (69.0)	26 (72.2)	
Rectum (%)	18 (18.0)	2 (5.6)	
Rectosigmoid (%)	9 (9.0)	2 (5.6)	
Sigmoid colon (%)	34 (34.0)	18 (50.0)	
Descending colon (%)	5 (5.0)	3 (8.3)	
Splenic flexure (%)	3 (3.0)	1 (2.8)	
Right sided (%)	31 (31.0)	10 (27.8)	
Transverse colon (%)	5 (5.0)	1 (2.8)	
Hepatic flexure (%)	6 (6.0)	1 (2.8)	
Ascending colon (%)	12 (12.0)	3 (8.3)	
Cecum (%)	8 (8.0)	5 (13.9)	
**Morphology**			**0.02**
Non-polypoid	66 (66.0)	31 (86.1)	0.04
Annular (%)	27 (27.0)	21 (58.3)	
Non-annular/saddle-shaped (%)	39 (39.0)	10 (27.8)	
Polypoid (%)	34 (34.0)	5 (13.9)	0.96
Is; sessile (%)	18 (18.0)	2 (5.6)	
Isp; semi-pedunculated (%)	8 (8.0)	1 (2.8)	
Ip; pedunculated (%)	7 (7.0)	2 (5.6)	
0-IIa; flat (%)	1 (1.0)	0 (0.0)	
**Luminal stenosis**			**0.015**
Present (%)	17 (17.0)	14 (38.9)	
Absent (%)	83 (83.0)	22 (61.1)	
**Dimensions**			
Median long axis, cm (IQR)	3.0 (2.1-3.9)	4.3 (3.2-5.3)	<0.001
Median thickness/short axis*, cm (IQR)	1.3 (0.9-1.8)	1.5 (1.2-1.9)	0.07
Median volume, cm^3^ (IQR)	9.1 (3.5-20.1)	23.2 (9.5-43.6)	0.001
**Conspicuity**			
Reader 1, median (IQR)	75.0 (25.0-86.3)	95.0 (79.5-100)	<0.001
Reader 2, median (IQR)	52.0 (25.0-64.0)	70.0 (44.3-75.0)	0.001

*For non-polypoid tumours, dimension given is tumour thickness. For polypoid lesions, dimension given is orthogonal short axis

#### Morphology, dimensions, and volume

Tumour morphology differed significantly between the two groups; screen-detected tumours were more likely to be polypoidal than symptomatic tumours (screen-detected: 34/100, 34.0 %; symptomatic: 5/36, 13.9 %, OR = 3.80, 95 %CI 1.26-11.49, p = 0.02, Table 1). The Paris classification of polypoidal tumours is shown in Table 1, and was not significantly different between the two groups. Considering only non-polypoidal tumours, there were significantly fewer annular cancers in the screening group (screen-detected:27/66, 40.9 %; symptomatic:21/31, 67.7 %, OR = 0.36, 95 %CI 0.13-0.94, p = 0.04). Screen-detected cancers were also significantly less likely to cause ≥50 % luminal stenosis (screen-detected:17/100, 17.0 %; symptomatic: 14/36, 38.9 %, OR = 0.33, 95 %CI 0.13-0.81, p = 0.015, Fig. 1). Data for individual radiologists are provided in Supplementary Table 2.

**Fig. 2:**
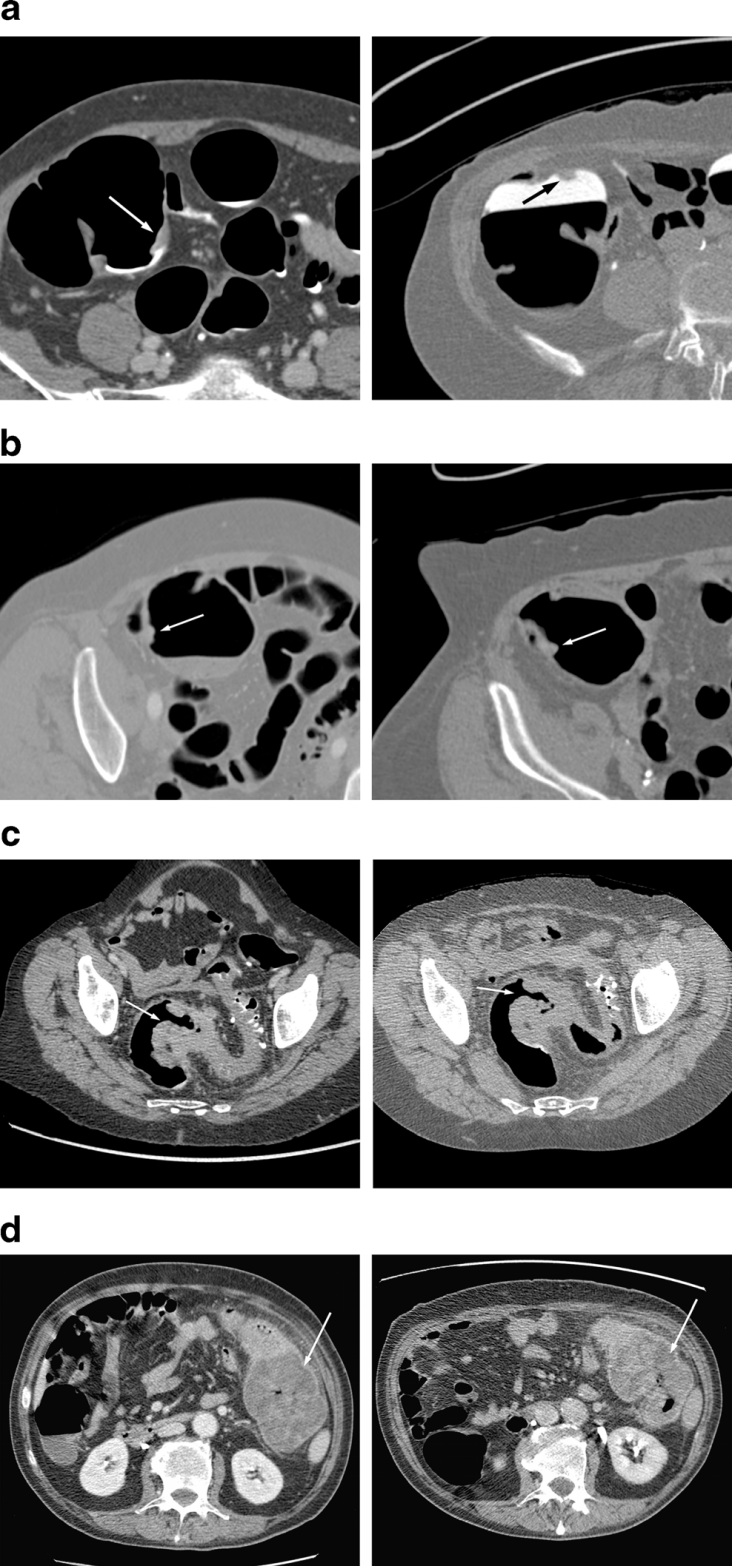
Examples of different cancers; subtle screening (**a**), subtle symptomatic (**b**), obvious screening (**c**), and obvious symptomatic (**d**) tumours (arrows). In each case, the left panel shows the supine image and the right panel shows the prone series. The subtle tumours were assigned a mean conspicuity score of 10 (screening case) and 12 (symptomatic case); the screening case (**a**) was not detected by CAD. The obvious tumours were assigned a mean conspicuity score of 97.5 (screening case) and 100 (symptomatic case)

Tumours in screening patients were also significantly smaller than those in symptomatic patients. Median long-axis dimension of screen-detected tumours (averaged between both radiologists) was 3.0 cm (IQR 2.1-3.9 cm), compared to 4.3 cm (IQR 3.2-5.3 cm) for symptomatic tumours (p < 0.001). Five (5.0 %) screen-detected cancers were less than 10 mm in long axis whereas none of the symptomatic cancers measured <10 mm. Tumour volume was also significantly lower for screen-detected tumours (median 9.1 cm^3^, IQR 3.5-20.1 cm^3^) compared to symptomatic CRC (median 23.2 cm^3^, IQR 9.5-43.6 cm^3^, p = 0.001, Table 1, Fig. 2). Tumours that were detected in the randomized trials, but for which discs were not available for inclusion in this study nonetheless had their lengths measured by site radiologists in the original trials; mean tumour length was measured as 5.2 cm (IQR 3.5-7.0 cm); see Supplementary Table 3.

#### Relative conspicuity

Screen-detected tumours were judged significantly less conspicuous than symptomatic tumours by both radiologists (Reader 1, screen-detected tumours: median 75.0, symptomatic: 95.0, p < 0.001; Reader 2, screen-detected: 52.0, symptomatic: 70.0, p = 0.001, Table 1, Fig. 1). Twelve tumours were assigned a conspicuity score of ≤10 by Reader 1; all were screen-detected cancers. Reader 2 scored 9 tumours as having a conspicuity of ≤10; all except one were screen-detected tumours, see Fig. 2.

#### Sensitivity of standalone CAD

CAD processing was successful for 91/98 (93 %) patients from the screening program, including both patients with two tumours, meaning there were 93 screen-detected tumours for analysis of CAD sensitivity. In total, 30/35 symptomatic patients (depicting 31 tumours) had successful CAD processing. We were unable to resolve the reason for CAD failure in the remaining cases.

**Fig. 3:**
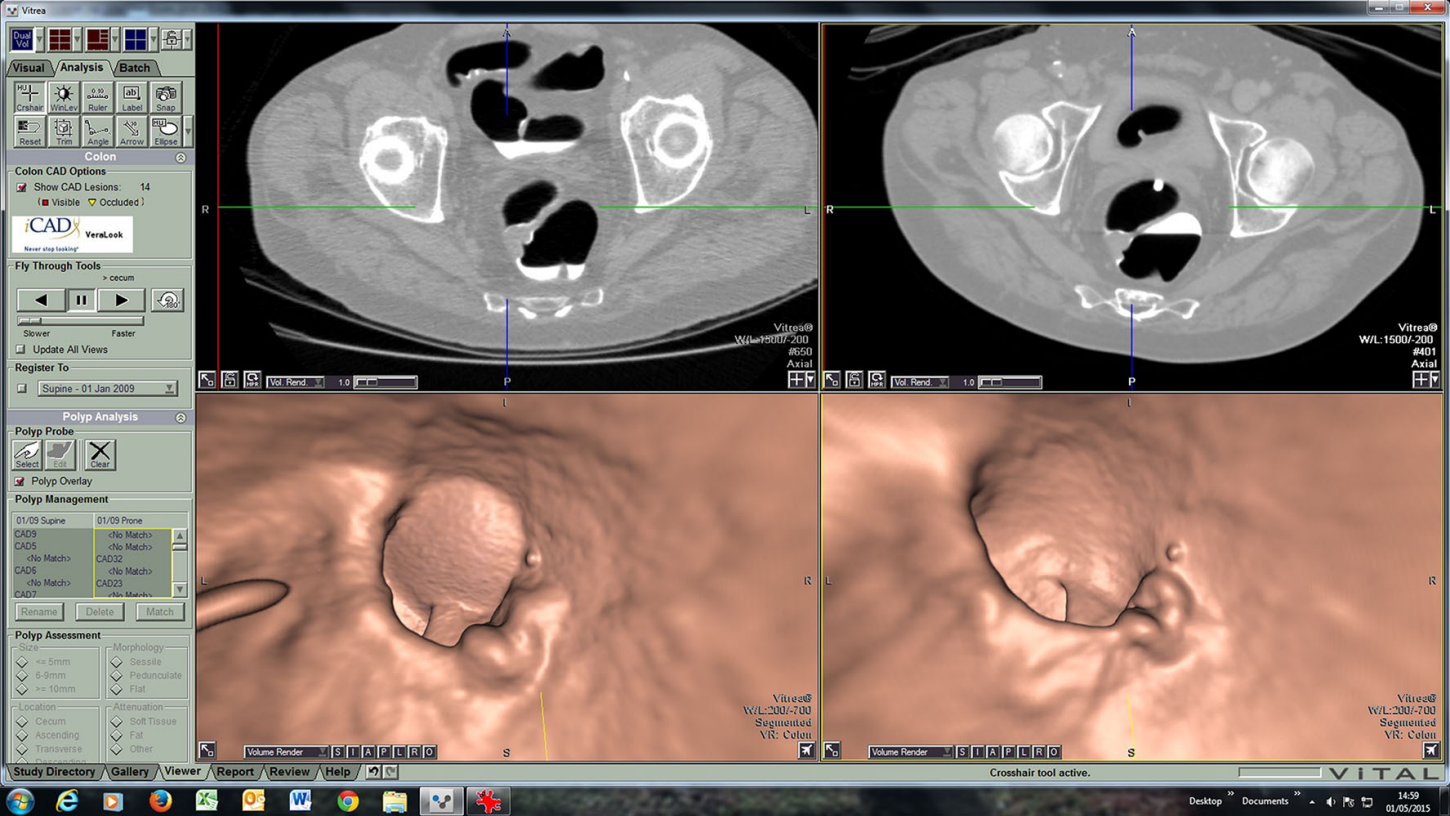
Example of an upper rectal tumour missed by CAD in a screening patient. Image quality was judged to be good by both readers, with a small amount of residual fluid which was well-tagged

The total number of CAD marks per patient was not significantly different between screening and symptomatic cases (screening: mean of 19.5 CAD marks/patient, range 4-32; symptomatic: 19.4 CAD marks/patient, range 6-46, p = 0.72). However, the proportion of cancers missed by CAD was significantly greater for screen-detected vs. symptomatic tumours. Specifically, the CAD system identified only 72/93 screen-detected tumours with at least one CAD mark, giving a standalone sensitivity of 77.4 % (95 %CI 67.9-84.7 %). Conversely, 30/31 symptomatic tumours were marked by CAD, giving a significantly higher sensitivity of 96.9 % (95 %CI 83.8-99.4 %, p = 0.02, Fig. 3).

There was no significant difference between location, morphology, or size of cancers that were missed by CAD when compared to those that CAD detected successfully (Table 2). However, the cancers that were missed by CAD were judged as significantly less conspicuous than those detected by CAD, by both readers (Reader 1, median conspicuity score of cancers missed by CAD = 21.0 (IQR 15.0-60.0) vs. 80.0 (IQR 53.8-94.5) for cancers detected, p < 0.001; Reader 2, median conspicuity of cancers missed by CAD = 36.0 (IQR 22.0-57.8) vs. 58.0 (IQR 40.0-68.8) for cancers detected, p = 0.01).

**Table 2:** Characteristics of cancers detected and missed by the CAD system. All percentages refer to the proportion of tumours within that detection category (i.e., detected or missed)

Number	Detected tumours (n = 102)	Missed tumours (n = 22)	p value
**Segmental location**			**0.44**
Left sided (%)	71 (69.6)	14 (63.6)	
Rectum (%)	15 (14.7)	4 (18.2)	
Rectosigmoid (%)	8 (7.8)	1 (4.5)	
Sigmoid colon (%)	37 (36.3)	9 (40.9)	
Descending colon (%)	7 (6.9)	0 (0.0)	
Splenic flexure (%)	4 (3.9)	0 (0.0)	
Right sided (%)	31 (30.4)	8 (36.4)	
Transverse colon (%)	4 (3.9)	1 (4.5)	
Hepatic flexure (%)	6 (5.9)	1 (4.5)	
Ascending colon (%)	10 (9.8)	4 (18.2)	
Cecum (%)	11 (10.8)	2 (9.1)	
**Morphology**			**0.89**
Non-polypoid	72 (70.6)	15 (68.2)	
Annular	38 (37.3)	6 (27.3)	
Saddle-shaped	34 (33.3)	9 (40.9)	
Polypoid	30 (29.4)	7 (31.8)	
Is; sessile (%)	17 (16.7)	3 (13.6)	
Isp; semipedunculated (%)	5 (4.9)	2 (9.1)	
Ip; pedunculated (%)	8 (7.8)	1 (4.5)	
0-IIa; flat (%)	0 (0.0)	1 (4.5)	
**Dimensions**			
Median long axis, cm (IQR)	3.3 (2.4-4.5)	2.9 (1.7-5.3)	0.74
Median short axis, cm (IQR)	1.3 (1.0-1.8)	1.2 (0.9-1.8)	0.31
Median volume, cm^3^ (IQR)	12.2 (4.9-27.8)	5.2 (1.5-35.5)	0.94
**Conspicuity**			
Reader 1, median (IQR)	80.0 (53.8-94.5)	21.0 (15.0-60.0)	<0.001
Reader 2, median (IQR)	58.0 (40.0-68.8)	36.0 (22.0-57.8)	0.01

#### TNM stage and prognostic group

There was “reasonable” to “good” agreement between radiologists for tumour stage (kappa = 0.77), presence of involved lymph nodes (kappa = 0.74) and overall prognostic category (kappa = 0.78). Agreement for the presence of macroscopic venous invasion was “moderate” (kappa = 0.54).

Screen-detected tumours were of significantly earlier radiological local stage than symptomatic tumours (OR = 0.17, 95 %CI 0.07-0.41, p < 0.001, Table 3). They were also significantly less likely to have radiologically involved lymph nodes (screening 28/100, 28.0 %; symptomatic: 20/36, 55.6 %; OR = 0.31, 95 %CI 0.13-0.72, p = 0.006) or macroscopic vascular invasion (screening: 13/100, 13.0 %; symptomatic: 12/36, 33.3 %,OR = 0.26, 95 %CI 0.10-0.67, p = 0.01). When considering the overall CT-derived prognostic group, screening patients were significantly less likely to have poor-prognosis tumours (screening: 24/100, 24.0 %;symptomatic: 21/36, 58.3 %, OR = 0.21, 95 %CI 0.09-0.50, p < 0.001). Data for individual radiologists are provided in Supplementary Table 2.

**Table 3:** Radiological tumour and nodal staging, according to the TNM 7th edition, presence of macroscopic vascular invasion, and overall CT-estimated tumour prognostic category, split by case origin (i.e., symptomatic vs. screening). All percentages use the number of tumours of that category as the denominator

	Screen-detected tumours (n = 100)	Symptomatic tumours (n = 36)	p value
**Tumour stage**			**<0.001**
T1	22 (22.0)	4 (11.1)	
T2	47 (47.0)	5 (13.9)	
T3	29 (29.0)	24 (66.7)	
T4	2 (2.0)	3 (8.3)	
**Nodal involvement**			**0.006**
Node negative	72 (72.0)	16 (44.4)	
Node positive	28 (28.0)	20 (55.6)	
**Vascular invasion**			**0.006**
Absent	87 (87.0)	24 (66.7)	
Present	13 (13.0)	12 (33.3)	
**Prognostic category**			**<0.001**
Good prognosis	76 (76.0)	15 (41.7)	
Poor prognosis	24 (24.0)	21 (58.3)	

## Discussion

CTC is employed frequently to diagnose CRC, both for patients with colorectal symptoms and for asymptomatic screenees. We found significant differences in morphology, size, volume, and frequency of luminal stenosis between screen-detected and symptomatic cancers. Furthermore, screen-detected cancers were of earlier radiological T-stage, were less likely to have involved lymph nodes or vascular invasion, and were less likely to meet CT criteria for poor prognosis. Screen-detected cancers were significantly less conspicuous than symptomatic CRC, and were less likely to be identified by a commercial CAD system. Most radiologists gather initial experience of CTC in symptomatic patients [27]. In this setting, the prevalence of abnormality is high, and we have shown here that significant abnormalities (i.e., CRC) are usually obvious. Conversely, screening with CTC is different; prevalence of abnormality is lower, and even when CRC is present, it may be very subtle. Therefore, radiologists embarking on screening CTC, even if they have considerable experience with symptomatic CTC, may need specific guidance, training, and quality assurance prior to reporting for a screening program.

CTC is highly sensitive for CRC, with two meta-analyses suggesting a sensitivity of 96 % [1, 2]. The CTC features of CRC are well-documented, with the majority of cancers being either large polyps or masses [16, 28]. However, few reports distinguish between screen-detected and symptomatic cancers. Regarding screening, a large USA series reported the findings of colonic and extracolonic malignancy in 10,286 asymptomatic individuals, describing 22 cases of CRC [16]. Although detailed morphological parameters of these 22 cancers were not provided, the authors noted that the majority were large (mean 3.3 cm) and appeared as “frankly invasive masses or malignant polyps”. Similarly, we found a median size for screen-detected CRC of 3.0 cm, although five cancers were subcentimeter and one case of invasive CRC was found in a 5-mm polyp.

We found significant differences in conspicuity between screening and symptomatic CRC. A substantial proportion of screen-detected tumours were judged as hard to detect, with 14 of 100 cases receiving a conspicuity score of ≤10 by at least one of the radiologists. Although the “average” or “typical” screen-detected cancer is relatively obvious (median conspicuity score > 50 for both radiologists), subtle cases of CRC are much more common in a screening population.

Not only were screen-detected CRC more difficult to detect by human readers, the CAD system that we used also had significantly poorer detection. CAD systems are primarily designed to detect polyps rather than CRC, although many cancers have polypoid features and so most CRC are detected by CAD [17]. While our findings agree with this observation for symptomatic patients (CAD sensitivity of 96.8 %), we found that the CAD system used here missed a significant proportion (22.6 %) of CRC in screenees. We doubt this is due to CTC data quality, since it was not significantly different between the two groups. Although fecal tagging was used for most of the screening cases, and not for the symptomatic cases, CAD systems are designed to operate under such conditions, including the system we used [29]. It is more plausible that screen-detected tumours have inherently different morphologic characteristics that render CAD less effective. We doubt that this is due to overall gross polyp morphology, since this was not significantly different between lesions detected and missed by CAD (Table 2). Instead, it seems likely that there are more subtle morphological differences between the two groups other than gross morphology. The cancers missed by CAD were also judged as subjectively more subtle than those detected, a finding that agrees with prior work evaluating the conspicuity of colorectal polyps (as opposed to established CRC) [30]. We only tested one specific CAD system; other algorithms may perform differently.

We found that screen-detected cancers were of an earlier radiological stage than CRC in symptomatic patients. This agrees with Pickhardt et al., who used screening CTC to detect CRC at a significantly earlier stage than the US average [16]. We compared radiological staging rather than histopathological staging since our primary purpose was to compare CTC appearances of screen-detected vs. symptomatic cancers; earlier histopathological stage of tumours detected by CTC following FOBt screening has been reported previously [31].

Our study has limitations. We recruited patients from an FOBt-based screening program and therefore, by definition, all patients had occult blood loss. However, it is unlikely that CRC in the general screening population would be larger than in FOBt-positive individuals, meaning that our primary conclusions regarding the relative size and conspicuity of screen-detected vs. symptomatic tumours (i.e., the former being smaller and more subtle) are likely generalizable. Furthermore, we included only those patients with no prior colonoscopy (to avoid the inevitable spectrum bias introduced by including patients with failed colonoscopy, many of whom will have only undergone CTC because of a colonoscopically-impassable tumour). There is a small chance that this biased our cohort toward particularly small or subtle cancers, although this seems unlikely.

Secondly, CTC examinations performed in the SIGGAR trials did not use fecal tagging, whereas approximately three-quarters of the EBCSP examinations did. However, this fact would tend to make symptomatic cancers more difficult to detect, the exact opposite of what we found. It is plausible that the differences in contemporary practice are even greater. Thirdly, conspicuity is necessarily a subjective measure, although we guarded against this by using more than one reader and employed measurements of tumour size/volume and CAD detection as more objective outcomes. Nonetheless, it is important to note that low conspicuity/lack of a CAD mark does not equate to a failure of detection; 90 % of the 80 CRC screening cases for which we were provided with radiological reports were diagnosed at the time of CTC interpretation (or, conversely, 10 % were missed), compared to 97 % of the symptomatic cases. Fourthly, we only evaluated these cases using a single CAD system; it is possible that other algorithms may have different performance. Although we collated a relatively large series of CRC for this study, not all CTC data sets could be retrieved for image review; it is possible that the cases that we reviewed do not represent the full spectrum of disease in symptomatic and screening settings. Finally, radiological assessment of both CRC TNM stage and prognostic category are imperfect, although interreader agreement in our study was good and compared favorably with existing literature [25, 26].

In summary, colorectal cancers detected by CTC in an FOBt screening program were significantly smaller, more subtle, and of earlier radiological stage than symptomatic tumours. They were also more likely to go undetected by CAD, which may be of relevance for CAD package design. Radiologists interpreting both screening and symptomatic CTC should be aware of the differences in morphology and conspicuity of CRC that distinguish the two patient populations.

## Electronic supplementary material

Below is the link to the electronic supplementary material.

Supplementary Table 1 (DOC 59 kb)

Supplementary Table 2 (DOC 53 kb)

Supplementary Table 3 (DOC 37 kb)
