# Supplementary material for: Appearances of screen-detected versus symptomatic colorectal cancers at CT colonography
Source: Eur Radiol. 2016 Apr 5;26(12):4313–22. doi: 10.1007/s00330-016-4293-7 (PMC5101282; doi:10.1007/s00330-016-4293-7)
Supplement: Supplementary file 1 — (DOC 59 kb) [file 330_2016_4293_MOESM1_ESM.doc]

## Supplementary Table 1 Image acquisition parameters for each site included in the study

| Center | Collimation | Reconstruction interval | kVp | Fecal tagging | Intravenous contrast |
| --- | --- | --- | --- | --- | --- |
| Sympt 1 | 1.0 | 1.0 | 120 | No | No |
| Sympt 2 | 2.5 | 1.25 | 120 | No | Yes |
| Sympt 3 | 2.5 | 1.25 | 120 | No | Yes |
| Sympt 4 | 1.0 | 1.0 | 120 | No | Yes |
| Sympt 5 | 2.5 | 1.25 | 120 | No | Yes |
| Sympt 6 | 3.0 | 1.5 | 120 | No | No |
| Sympt 7 | 2.5 | 1.25 | 120 | No | No |
| Sympt 8 | 3.0 | 1.0 | 120 | No | Yes |
| Sympt 9 | 2.5 | 1.25 | 120 | No | Yes |
| Sympt 10 | 1.0 | 1.0 | 120 | No | Yes |
| Screen 1 | 0.9 | 0.7 | 120 | Yes | No |
| Screen 2 | 0.625 | 0.625 | 120 | Yes | Yes |
| Screen 3 | 1.25 | 1.25 | 120 | Yes | Yes |
| Screen 4 | 1.0 | 0.7 | 120 | Yes | Yes |
| Screen 5 | 1.25 | 1.25 | 120 | Yes | Yes |
| Screen 6 | 1.0 | 1.0 | 120 | No | Yes |
| Screen 7 | 0.75 | 0.75 | 100 | Yes | Yes |
| Screen 8 | 2.0 | 2.0 | 120 | Yes | No |
| Screen 9 | 0.6 | 2.0 | 120 | Yes | No |
| Screen 10 | 0.75 | 0.75 | 120 | Yes | No |
| Screen 11 | 2.0 | 1.0 | 120 | Yes | No |
| Screen 12 | 1.2 | 1.0 | 100 | Yes | No |
| Screen 13 | 1.25 | 1.0 | 120 | No | Yes |
| Screen 14 | 0.625 | 1.25 | 120 | Yes | Yes |
| Screen 15 | 1.0 | 1.0 | 120 | Yes | Yes |
| Screen 16 | 1.5 | 1.5 | 120 | Yes | No |
| Screen 17 | 2.5 | 1.25 | 120 | No | Yes |
| Screen 18 | 1.0 | 1.0 | 120 | Yes | Yes |
| Screen 19 | 0.625 | 0.625 | 120 | Yes | Yes |
| Screen 20 | 1.0 | 2.0 | 120 | Yes | No |
| Screen 21 | 2.0 | 2.0 | 120 | No | No |
| Screen 22 | 1.25 | 1.25 | 120 | Yes | No |
| Screen 23 | 0.625 | 0.625 | 120 | No | No |
| Screen 24 | 0.75 | 0.75 | 120 | Yes | No |
| Screen 25 | 1.0 | 1.0 | 120 | Yes | No |
