# Supplementary material for: Appearances of screen-detected versus symptomatic colorectal cancers at CT colonography
Source: Eur Radiol. 2016 Apr 5;26(12):4313–22. doi: 10.1007/s00330-016-4293-7 (PMC5101282; doi:10.1007/s00330-016-4293-7)
Supplement: Supplementary file 2 — (DOC 53 kb) [file 330_2016_4293_MOESM2_ESM.doc]

**Supplementary Table 2** Morphology and dimensions ­of symptomatic and screen-detected cancers, split by radiologist. Percentages use the number of tumors of that category (i.e., symptomatic or screening) as the denominator

|  | |  | |  | **Screen-detected tumors (n=100)** | | **Symptomatic tumors (n=36)** | |
| --- | --- | --- | --- | --- | --- | --- | --- | --- |
| **Morphology** | | | | | Radiologist 1 | Radiologist 2 | Radiologist 1 | Radiologist 2 |
|  | | Non-polypoid | | | 63 (63.0) | 60 (60.0) | 28 (77.8) | 31 (86.1) |
|  | | Annular (%) | | | 25 (25.0) | 27 (27.0) | 21 (58.3) | 20 (55.6) |
|  | | Non-annular/saddle-shaped (%) | | | 38 (38.0) | 33 (33.0) | 7 (19.4) | 11 (30.6) |
|  | | Polypoid (%) | | | 37 (37.0) | 40 (40.0) | 8 (22.2) | 5 (13.9) |
|  | |  | Is; sessile (%) | | 18 (18.0) | 24 (24.0) | 4 (11.1) | 3 (8.3) |
|  | |  | Isp; semi-pedunculated (%) | | 9 (9.0) | 10 (10.0) | 2 (5.6) | 1 (2.8) |
|  | |  | Ip; pedunculated (%) | | 7 (7.0) | 3 (3.0) | 2 (5.6) | 1 (2.8) |
|  | |  | 0-IIa; flat (%) | | 3 (3.0) | 2 (2.0) | 0 (0.0) | 0 (0.0) |
| **Luminal stenosis** | | | | | | | | |
|  | | Present (%) | | | 14 (14.0) | 19 (19.0) | 14 (38.9) | 15 (41.7) |
|  | | Absent (%) | | | 86 (86.0) | 81 (81.0) | 22 (61.1) | 21 (58.3) |
| **Dimensions** | | | | | | | | |
|  | Median long axis, cm (IQR) | | | | 3.1 (2.2 to 3.8) | 3.0 (1.9 to 4.1) | 4.0 (3.3 to 5.5) | 4.3 (3.2 to 5.4) |
|  | Median thickness/short axis*, cm (IQR) | | | | 1.1 (0.9 to 1.6) | 1.4 (1.1 to 1.9) | 1.4 (1.1 to 1.8) | 1.5 (1.2 to 2.1) |
| **Tumor stage** | | | | | | | | |
|  | | T1 | | | 22 (22.0) | 30 (30.0) | 4 (11.1) | 3 (8.3) |
|  | | T2 | | | 47 (47.0) | 40 (40.0) | 8 (22.2) | 8 (22.2) |
|  | | T3 | | | 30 (30.0) | 28 (28.0) | 23 (63.9) | 20 (55.6) |
|  | | T4 | | | 1 (1.0) | 2 (2.0) | 1 (2.8) | 5 (13.9) |
| **Nodal involvement** | | | | | | | | |
|  | | Node negative (%) | | | 71 (71.0) | 63 (63.0) | 17 (47.2) | 12 (33.3) |
|  | | Node positive (%) | | | 29 (29.0) | 27 (27.0) | 19 (52.8) | 24 (66.7) |
| **Vascular invasion** | | | | | | | | |
|  | | Absent (%) | | | 87 (87.0) | 88 (88.0) | 25 (69.4) | 24 (66.7) |
|  | | Present (%) | | | 13 (13.0) | 12 (12.0) | 11 (30.6) | 12 (33.3) |
| **Prognostic category** | | | | | | | | |
|  | | Good prognosis (%) | | | 78 (78.0) | 75 (75.0) | 15 (41.7) | 14 (38.9) |
|  | | Poor prognosis (%) | | | 22 (22.0) | 25 (25.0) | 21 (58.3) | 22 (61.1) |
