# Supplementary material for: Appearances of screen-detected versus symptomatic colorectal cancers at CT colonography
Source: Eur Radiol. 2016 Apr 5;26(12):4313–22. doi: 10.1007/s00330-016-4293-7 (PMC5101282; doi:10.1007/s00330-016-4293-7)
Supplement: Supplementary file 3 — (DOC 37 kb) [file 330_2016_4293_MOESM3_ESM.doc]

## Supplementary Table 3 Tumor stage, location, diagnostic confidence scores, and mean dimensions assigned by original reporting radiologists for the tumors diagnosed in symptomatic patients, but for whom CTC images were not available

| Tumor stage | | |
| --- | --- | --- |
|  | T1 | 1 |
|  | T2 | 12 |
|  | T3 | 29 |
|  | T4 | 16 |
|  | Not recorded | 4 |
| Tumor location | | |
|  | Rectum | 9 |
|  | Rectosigmoid | 11 |
|  | Sigmoid colon | 20 |
|  | Descending colon | 0 |
|  | Transverse colon | 7 |
|  | Ascending colon | 7 |
|  | Caecum | 8 |
| Diagnostic confidence | | |
|  | Excellent | 40 |
|  | Good | 7 |
|  | Poor | 2 |
|  | Not recorded | 13 |
| Tumor dimensions (cm) | | |
|  | Mean | 5.2cm |
|  | Interquartile range | 3.5 to 7.0cm |
